# P-1762. The Host Transcriptional Response to Fungal Infection Suggests Disturbances of the Innate Immune System and Cytokine Signaling in Hematologic Malignancy that Increase Susceptibility to Infection Relative to Hematopoietic Stem Cell Transplantation

**DOI:** 10.1093/ofid/ofaf695.1933

**Published:** 2026-01-11

**Authors:** Julie M Steinbrink, Cameron Miller, Kelly Stanly, Barbara D Alexander, Micah T McClain

**Affiliations:** Duke University Medical Center, Durham, NC; Duke University Medical Center, Durham, NC; Duke University Medical Center, Durham, NC; Duke University School of Medicine, Durham, North Carolina; Duke University, Durham, North Carolina

## Abstract

**Background:**

Hematologic malignancy (HM) patients have an increased incidence of invasive fungal disease (IFD) (12% prior to anti-mold prophylaxis, with a decrease to 5% with prophylaxis), compared to hematopoietic stem cell transplant (HSCT) recipients (about 4%). Analysis of gene expression patterns (‘signatures’) in leukocytes can provide supplementary diagnostic and immunologic information about the variable and often dysregulated immune responses that occur during these devastating infections, even in the setting of neutropenia.

**Figure:**
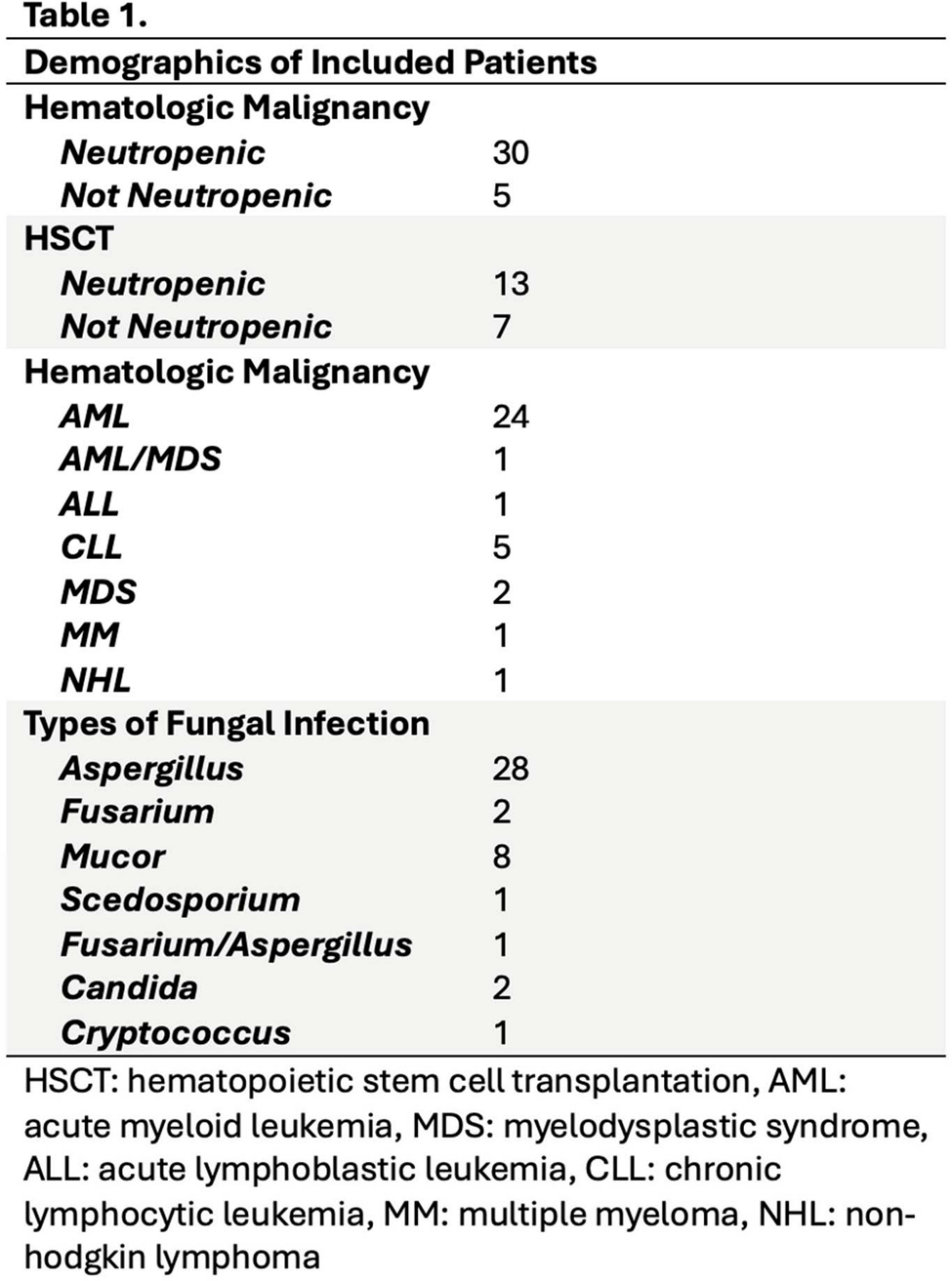

**Methods:**

Bulk RNA sequencing was performed from peripheral blood of neutropenic and non-neutropenic HM and HSCT recipients with adjudicated IFD. Differential expression (DEA) and over-representation analyses (ORA) were performed to identify and characterize transcriptional changes.

**Results:**

43 of 55 included subjects were neutropenic (Table 1). DEA revealed a coordinated decrease in expression in HM patients compared to HSCT patients for a subset of genes, and ORA was used to elucidate their potential etiology.

ORA showed out of 6,812 gene sets, 35 were significantly overrepresented among the gene subset. These overrepresented gene sets coalesced into large immune-related signals. These signals included: 1) inflammatory and innate immune responses; 2) leukocyte mediated immunity; 3) leukocyte migration, neutrophil chemotaxis; and 4) cytokine production. Individual genes most differentially expressed between the 2 infected groups included those encoding C-type lectins, TLR2, and other elements of antifungal immunity.

**Conclusion:**

We found that the immune responses (as demonstrated by their transcriptional profiles) of HM and HSCT patients to IFD differ in ways not explained by neutropenia alone. HM patients demonstrate differential expression of pattern recognition receptors and comparatively decreased innate immune responses and decreased cytokine production, potentially reflective of the prolonged disturbance of the innate immune system in HM patients compared to HSCT. This provides novel insight into the host response to real-world IFD in neutropenic patients, and the particular susceptibilities of HM patients to IFD.

**Disclosures:**

Julie M. Steinbrink, MD, MHS, Biomeme: patents for gene expression classifiers of fungal infection|McGraw Hill Publishing: royalties Micah T. McClain, MD, PhD, Biomeme: patents for gene expression classifiers of fungal infection|Darwin Biosciences: Board Member|UpToDate: Advisor/Consultant

